# BxPC3 pancreatic cancer cells express a truncated Smad4 protein upon PI3K and mTOR inhibition

**DOI:** 10.3892/ol.2014.1833

**Published:** 2014-01-28

**Authors:** ONICA LEGENDRE, AYISHA SOOKDEO, DAVID A. FOSTER

**Affiliations:** Department of Biological Sciences, Hunter College of The City University of New York, New York, NY 10065, USA

**Keywords:** Smad4, pancreatic cancer, chromosomal instability, BxPC3 cells, transforming growth factor-β

## Abstract

Smad4 is a critical regulator of transforming growth factor (TGF)-β signaling and is defective in numerous human cancers. In total, 30% of pancreatic cancers harbor a homozygous deletion of Smad4. The human pancreatic cancer cell line, BxPC3, has been reported to be Smad4-null due to a homozygous deletion and has been widely used as a Smad4-null model. The present study reports that Smad4 DNA is present in BxPC3 cells, and under conditions of suppressed mammalian target of rapamycin complex 1 (mTORC1) and phosphatidylinositol-3-kinase, a truncated Smad4 protein is expressed. While a high level of Smad4 protein can be expressed in these cells, the cells do not respond to TGF-β. The Smad4 defect in BxPC3 cells likely occurs via translocation rather than deletion as previously reported.

## Introduction

Smad proteins are intracellular mediators of transforming growth factor-β (TGF-β) and bone morphogenetic protein signaling pathways necessary for the regulation of a variety of critical processes, including embryonic development, fibrosis, tumor development, immune function and wound healing (1,2). Genomic sequencing has revealed numerous defects in the TGF-β signaling pathway of human pancreatic cancers (3). Smad4, also known as DPC4 (deleted in pancreatic carcinoma, locus 4), was first isolated and identified in pancreatic cancer on human chromosome 18q21.1 (4). Hahn *et al* reported that ~90% of human pancreatic cancers show allelic loss at chromosome 18q. Deletion of chromosome 18q, which encompasses the Smad4 region, significantly affects the prognosis of pancreatic cancer (4). Individuals with pancreatic cancer positive for Smad4 have shown a higher survival rate compared with those with pancreatic cancers negative for Smad4 (5). These findings indicate that the presence of Smad4 is critical in the development and treatment of human pancreatic cancers.

Chromosomal deletions occur in a number of cancers causing either the absence of the gene or loss of its function (6). According to the National Human Genomic Research Institute and the National Center for Biotechnology Information, translocation is defined as a type of chromosomal abnormality in which a chromosome breaks and a portion of it reattaches to a different chromosome. Studies have shown that >90% of human cancers possess a certain type of clonal cytogenetic change (6–8). The most widely described chromosomal abnormality involving chromosomal translocation is the Philadelphia chromosome (9,10). Produced from the fusion of chromosome 9 and a truncated chromosome 22, the Philadelphia chromosome leads to an oncogenic BCR-ABL gene, which is responsible for the development of chronic myelogenous leukemia (11–14). Thus, chromosomal translocations can lead to the formation of new genes caused by the merging or ablation of existing genes that contribute to the oncogenic phenotype.

Characterizing translocated genes involves the use of banding techniques originally described by Rowley in 1973 (10). However, studies used to identify homozygous deletions on chromosome 18q in pancreatic cancer cells utilized PCR-based assays that focused mainly on characterizing a specific region of chromosome 18q where homozygous deletions commonly occur, 18q21.1 (4,6). While deletion mapping can identify missing areas of a chromosome, in the case of Smad4 in BxPC3 cells, it does not take into consideration deletion due to translocation. While investigating the synthetic lethal interactions between the inhibition of as mammalian target of rapamycin complex 1 (mTORC1) and TGF-β signaling pathways in Smad4-null BxPC3 cells, the expression of a Smad4-like protein was identified. The present study therefore investigated whether the Smad4 gene is actually present in BxPC3 cells, a pancreatic cancer cell line widely used to represent a Smad4-null genotype.

## Materials and methods

### Cell lines and cell culture conditions

The BxPC3 and Panc1 cells used in this study were obtained from the American Type Culture Collection (ATCC, Manassas, VA, USA) and were maintained in Roswell Park Memorial Institute (RPMI) medium and Dulbecco’s modified Eagle’s medium (DMEM), respectively, supplemented with 10% fetal bovine serum (Hyclone, Waltham, MA, USA). BxPC3 cells were also obtained from a stock maintained by Dr Murray Korc (University of Indiana, Indianapolis, IN, USA). For transfection of siRNA, the cells were plated at a density of 10^5^ cells/60-mm plate 24 h prior to transfection. All transfections were performed using Lipofectamine 2000 (Gibco-BRL, Carlsbad, CA, USA) according to the manufacturer’s instructions.

### Materials

Rapamycin was obtained from LC Laboratories (Woburn, MA, USA). The phosphatidylinositol-3-kinase (PI3K) inhibitor, LY294002, and Wortmannin were obtained from Cell Signaling Technology, Inc. (Danvers, MA, USA). Total Smad2 (product number 5339S, monoclonal rabbit IgG), total Smad3 (product number 9523P, monoclonal rabbit IgG) and Smad4 (product number 9515, monoclonal rabbit IgG) primary antibodies were obtained from Cell Signaling Technology, Inc. (Danvers, MA, USA), and the glyceraldehyde 3-phosphate dehydrogenase antibody was obtained from Santa Cruz Biotechnology Inc. (Santa Cruz, CA, USA). Smad4 siRNA and non-targeted negative control siRNA duplexes were obtained from Santa Cruz Biotechnology Inc. Smad4 primers were designed and synthesized by IDT (Coralville, IA, USA). A DNeasy Blood and Tissue kit was obtained from Qiagen (Hilden, Germany) and a Phusion High Fidelity DNA Polymerase kit was obtained from New England Biolabs (Ipswich, MA, USA).

### Western blot analysis

Proteins were extracted from cultured cells in modified radioimmunoprecipitation assay buffer (Upstate Biotechnology Inc., Lake Placid NY, USA). Equal amounts of protein were subjected to SDS-PAGE separating gels. Electrophoresed proteins were then transferred to nitrocellulose and subjected to western blot analysis as described previously (15). The results of the western blot analysis were quantified using ImageJ software version 1.47 (NIH, Bethesda, MD, USA)

### PCR

Genomic DNA was extracted from the cells using the Qiagen DNeasy Blood and Tissue kit. PCR amplification was performed using primers specific to Smad4 DNA and the Phusion High Fidelity DNA Polymerase kit was used to determine the presence of Smad4 in each cell line. Exon 1: Forward primer, 5′-ATGCTCAGTGGCTTCTCGACAAGTTG-3′ and reverse primer, 3′-GGGCTTTTTAAAGCCTCTGCACCAG-5′. Exon 2: forward primer, 5′-CCTTGCAACGTTAGCTGT TGT-3′ and reverse primer, 3′-TGAAGCCTCCCATCCAATGTT CTC-5′. Exon 12: forward primer, 5′-GTTGATGTGGATACT TTTCACACCG-3′ and reverse primer, 3′-CTACCACAAAGC TGGCCTCTACCA-5′.

### Smad4 siRNA duplexes

Smad4 siRNA (human) is a pool of three different siRNA duplexes: Exon from nucleic acids 788-962, with sense, GCAUCGACAGAGACAUACAtt, and antisense, UGUAUGUCUCUGUCGAUGCtt. Exon from nucleic acids 2277-3086, with sense, GAUGACUGUUGAUGA AGUAtt, and antisense, UACUUCAUCAACAGUCAUCtt. Exon from nucleic acids 2277-3086, with sense, CAAGGUUGGUUGCUAAGAAtt and antisense, UUCUUA GCAACCAACCUUGtt. All sequences are provided in 5′→3′ orientation.

## Results

### PI3K and mTOR complex 1 (mTORC1) inhibition induces expression of Smad4 in BxPC3 cells

**Figure 1:**
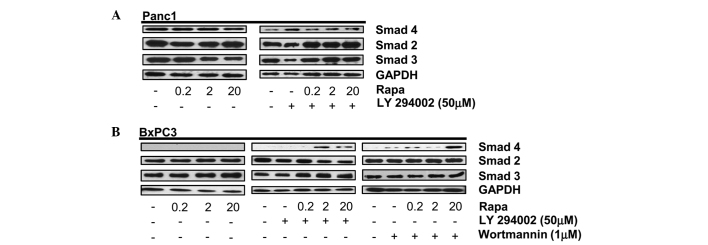
PI3K and mTORC1 inhibition induces expression of Smad4 in BxPC3 cells. (A) Panc-1 cells were plated at 10^5^ cells/60-mm plate in 10% serum. (A) After 24 h, the cells were shifted to media containing 10% serum with the indicated concentration of rapamycin (Rapa) (A, left panel). After another 4 h, the levels of Smad4, Smad2, Smad3 and GAPDH were analyzed by western blotting. Panc-1 cells were plated at 10^5^ cells/60-mm plate in 10% serum. After 24 h, the cells were shifted to media containing 10% serum and LY294002 (50 μM) for 1 h (A, right panel). The cells were then treated with the indicated concentration of Rapa. After another 4 h, the levels of Smad4, Smad2, Smad3 and GAPDH were analyzed by western blotting. (B) BxPC3 cells were plated as above and treated with the indicated concentrations of Rapa for 4 h (left panel) or LY294002 (50 μM; middle panel) or Wortmannin (1 μM; right panel) for 1 h. Cells initially treated with LY294002 or Wortmannin were then treated with the indicated concentration of Rapa. After 4 h, the levels of Smad4, Smad2, Smad3 and GAPDH were analyzed by western blotting. Western blots are representative of at least three independent experiments. PI3K, phosphatidylinositol-3-kinase; GAPDH, glyceraldehyde 3-phosphate dehydrogenase; mTORC1, mammalian target of rapamycin complex 1.

Defects in TGF-β signaling have been reported for the majority of pancreatic cancers, with deletions or mutations in Smad4 being most prevalent (3,4). Smad4 is a member of the Smad family and plays a pivotal role in mediating the downstream effects of the TGF-β signaling pathway (16,17). As a tumor suppressor, Smad4 regulates TGF-β-mediated epithelial cell growth or inhibition (18). Rapamycin has been reported to activate TGF-β signaling by mediating the nuclear translocation of activated Smad2/3 in complex with Smad4, while having no effect on the total protein levels (19). Rapamycin has also been reported to induce a negative feedback loop that activates the PI3K signaling pathway, which can block the expression and activation of Smad3, thus inhibiting the TGF-β signaling pathway (20). The present study therefore aimed to determine the effect of rapamycin on Smad2, Smad3 and Smad4 protein levels in Panc1 and BxPC3 cells. Data shown in Fig. 1A indicate that rapamycin alone or in combination with the PI3K inhibitor, LY294002, does not affect the expression levels of Smad2, 3 or 4 in Smad4 wild-type Panc1 cells. Sole treatment with rapamycin also does not affect the Smad2, 3 or 4 protein levels in BxPC3 cells; expression of Smad4 protein was not expected given the previously reported absence of Smad4 DNA in BxPC3 cells (4). As expected, Smad4 protein was not detected in the BxPC3 cells (Fig. 1B, left panel). However, upon dual treatment with rapamycin and the PI3K inhibitor, LY294002, or Wortmannin in the BxPC3 cells, a protein recognized by a Smad4 primary antibody was expressed (Fig. 1B, middle and right panels). This treatment had no effect on the level of Smad2 or Smad3 in the BxPC3 cells. These data indicate that under conditions where mTORC1 and PI3K signaling pathways are inhibited, a protein containing a similar amino acid sequence to that of wild-type Smad4 is expressed in BxPC3 cells.

### siRNA for Smad4 decreases the Smad4-like protein in BxPC3 cells

**Figure 2:**
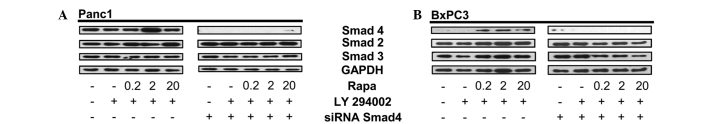
siRNA for Smad4 decreases the Smad4-like protein in BxPC3 cells. (A) Panc-1 cells were plated in media containing 10% serum for 24 h. The cells were then transfected with either control or Smad4 siRNA. After 48 h, the Panc1 cells were placed in fresh media containing 10% serum and LY294002 (50 μM) for 1 h, then rapamycin (Rapa; 20 μM) for 4 h at the indicated concentrations. The total lysate was analyzed by western blotting for levels of Smad4, Smad2, Smad3 and GAPDH. Western blots are representative of at least two independent experiments. (B) BxPC3 cells were plated in media containing 10% serum for 24 h. The cells were then transfected with either control or Smad4 siRNA. After 24 h, the BxPC3 cells were placed in fresh media containing 10% serum and LY294002 (50 μM) for 1 h, then Rapa (20 μM) for 4 h at the indicated concentrations. The cells were then collected, lysed and analyzed by western blotting for levels of Smad4, total Smad2, total Smad3 and GAPDH. Western blots are representative of at least two independent experiments. GAPDH, glyceraldehyde 3-phosphate dehydrogenase.

**Figure 3:**
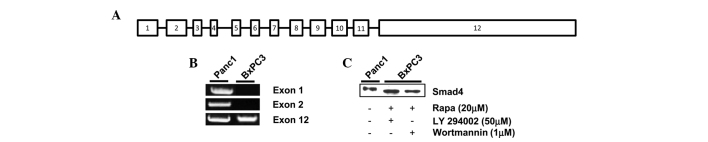
Smad4 DNA is amplified in exon 12 of BxPC3 pancreatic cancer cells. (A) Schematic of Smad4 gene. (B) Panc1 and BxPC3 cells were plated at a density of 10^5^ cells/60-mm plate. After 24 h, the cells were provided with fresh media containing 10% serum, and DNA from the Panc1 and BxPC3 cells was extracted using a Quigen DNA extraction kit. The acquired DNA was analyzed by PCR. PCR analyses are representative of at least two independent experiments. (C) BxPC3 cells were plated as stated above and then shifted to media containing 10% serum and LY294002 (50 μM) or Wortmannin (1 μM) for 1 h. The cells were then treated with the indicated concentration of rapamycin (Rapa). After 4 h, the levels of Smad4 were analyzed by western blotting. Panc-1 was used as a positive control.

The present study showed that Smad4 is detected under stress conditions in BxPC3 cells (Fig. 1). To further confirm that the protein expressed upon dual inhibition of the mTORC1 and PI3K signaling pathways in BxPC3 cells is Smad4, Panc1 and BxPC3 cells were pretreated with Smad4 siRNA followed by dual treatment with rapamycin and LY294002. Data in Fig. 2 show that Smad4 siRNA inhibits the expression of Smad4 in Panc1 cells (Fig. 2A), as well as the stress-induced Smad4-like protein in BxPC3 cells (Fig. 3B). The human Smad4 siRNA is a pool of three different siRNA duplexes, one from nucleic acids 788-962 (exon 3) and two from nucleic acids 2277-3086 (exon 12). Therefore, this data indicates that the abrogated expression of Smad4 in BxPC3 cells is linked to the siRNA duplexes that target either the exon 3 or 12 region of Smad4 DNA. Repeat experiments performed on the BxPC3 cells obtained from Dr Murray Korc showed the same pattern (data not shown).

### Smad4 DNA is amplified in exon 12 of BxPC3 pancreatic cancer cells

Proteins are generated via transcription and then translation from the subsequent DNA sequence. Therefore, proteins cannot be made without the presence of the corresponding DNA sequence. It has been previously reported that Smad4 DNA is amplified in Panc1 but not BxPC3 cells (4,21). It is important to note that Smad4 DNA contains 12 exons, which encode for the 552-amino acid Smad4 protein; exon 12 being the largest, measuring 6.787 kb (Fig. 3A) (22). Therefore, to determine the source of the inducible Smad4 protein in the BxPC3 cells, DNA extracted post-treatment with rapamycin and LY294002 was analyzed by PCR. Exon 1 of Smad4 has been previously used to identify the presence or absence of the gene (4). As shown in Fig. 3B, DNA was amplified in the Panc1 cells for exon 1, 2 and 12, the largest primer derived exons, in the presence of serum. However, when analyzing the BxPC3 cells, a DNA amplification band was only observed for exon 12 (Fig. 3B). Given that only the Smad4 DNA fragment corresponding to exon 12 is present in BxPC3 cells, it is possible that the protein expressed post-treatment with rapamycin and the PI3K inhibitors is a truncated form of Smad4. As with the Philadelphia chromosome, depending on the precise translocated region of the Smad4 gene, the molecular weight of the corresponding protein can be altered (14). Therefore, to determine the relative size of Smad4 expressed in the BxPC3 cells, the cells that were dually treated with high-dose rapamycin and LY294002 or Wortmannin were analyzed for Smad4 on the same western blot gel using Panc-1 cells as a positive control. As shown in Fig. 3C, Smad4 in the BxPC3 cells had a slightly faster electrophoretic mobility than wild-type Panc-1 Smad4. Taken together, these data indicate that BxPC3 cells express an inducible truncated version of the Smad4 protein, encoded mostly in exon 12 of Smad4 DNA.

## Discussion

BxPC3 cells are reported to have a homozygous deletion on chromosome 18q, which encompasses the coding region for Smad4/DPC4, 18q21.1 (4). PCR-assays, along with the combination of deletion and physical mapping, were employed to identify the homozygous deleted regions of chromosome 18q and resulted in the identification of Smad4 as a tumor suppressor located at chromosome 18q21.1 (4,6). Hahn *et al* reported that chromosomal region 18q21.1 is deleted in 30% of pancreatic cancers, including in BxPC3 cells. Homozygous deletion of Smad4 has been correlated with the loss of expression of the corresponding protein (23). Smad4 plays a pivotal role in regulating TGF-β signaling and can function as a tumor suppressor or promoter (5,24).

The homozygous loss of Smad4 has been the basis for use of BxPC3 cells as a model for pancreatic cancer with defective TGF-β signaling. We previously reported that TGF-β was unable to rescue BxPC3 cells from rapamycin-induced cell death, as was the case in the Smad4 wild-type Panc1 pancreatic cancer cells (15) indicating that BxPC3 pancreatic cancer cells are not responsive to TGF-β. However, in the present study, we suggest that the basis for defective TGF-β signaling in BxPC3 cells is not due to the homozygous deletion of Smad4, in that much of the Smad4 gene is present and expressed in response to the stress of mTORC1 and PI3K suppression. Thus, it is more likely that the loss of the Smad4 gene on chromosome 18q is due to a translocation rather than homozygous deletion. Whether the truncated Smad4 protein has any phenotypic impact on BxPC3 cells is not known, however, it appears that BxPC3 cells do not respond to TGF-β.
